# Leveraging ectopic Hsp90 expression to assay the presence of tumor cells and aggressive tumor phenotypes in breast specimens

**DOI:** 10.1038/s41598-017-17832-x

**Published:** 2017-12-13

**Authors:** Brian Crouch, Helen Murphy, Stella Belonwu, Amy Martinez, Jennifer Gallagher, Allison Hall, Mary Scott Soo, Marianne Lee, Philip Hughes, Timothy Haystead, Nirmala Ramanujam

**Affiliations:** 1 Department of Biomedical Engineering, Duke University, Durham, NC 27710 USA; 2 Duke University Trinity College of Arts and Sciences, Durham, NC 27710 USA; 3 Department of Surgery, Duke University Medical Center, Durham, NC 27710 USA; 4 Department of Pathology, Duke University Medical Center, Durham, NC 27710 USA; 5 Department of Radiology, Duke University Medical Center, Durham, NC 27710 USA; 6 Department of Pharmacology and Cancer Biology, Duke University Medical Center, Durham, NC 27710 USA

## Abstract

Hsp90 has been studied extensively as a therapeutic target in breast cancer in pre-clinical and clinical trials, demonstrating a variety of roles in metastatic progression. The evidence to date suggests a compelling opportunity to leverage attributes of Hsp90 expression beyond therapeutics with potential applications in breast cancer diagnosis, prognosis, and recurrence risk assessment. In this study, we developed a completely non-destructive strategy using HS-27, a fluorescently-tethered Hsp90 inhibitor, to assay Hsp90 expression on intact tissue specimens with comparable contrast to *in vivo* administration routes, and demonstrate the feasibility of our approach in breast cancer patients. In addition to Hsp90 inhibition being most effective in glycolytic tumors, we found ectopic Hsp90 expression to be highest in glycolytic tumors reinforcing its role as an indicator of aggressive disease. This work sets the stage for immediately using Hsp90 to improve outcomes for breast cancer patients without affecting traditional care pathways.

## Introduction

Heat-shock protein 90 (Hsp90) is a molecular chaperone protein that helps other proteins (referred to as ‘clients’) fold and remain stable in their active conformation^1–3^. Many functions of Hsp90 are dependent on its N-terminal ATPase activity, and blocking ATP binding leads to loss of Hsp90 function and degradation via the proteasome^1^. A host of Hsp90 clients include oncoproteins, which regulate key ‘Hallmarks of Cancer’^3–5^, including cell proliferation, glycolytic metabolism, and evasion of cell death. Not surprisingly, increased Hsp90 expression is associated with poor prognosis and decreased survival in human breast cancer^6,7^.

Hsp90 is expressed in a cyclical fashion in breast cancer, where the cytosolic pool of Hsp90 is trafficked to the cell surface and then re-internalized^8^. Hsp90 expressed on the surface of breast cancer and melanoma cancer cells plays a role in increasing cell motility and promoting metastasis^9–13^ through chaperoning and activation of matrix metalloproteinase 2 (MMP2), a protein responsible for breaking down the extracellular matrix surrounding cancer cells^14,15^. Inhibition of extracellular Hsp90 significantly impedes cell motility and reduces incidence of metastasis in breast cancer models^13^. The intracellular pool of Hsp90 helps stabilize and activate proteins responsible for the formation of filopodia and lamellipodia in breast cancer, which promote cellular migration, an early step in the metastatic pathway^16^.

A wide variety of proteins involved in metabolism are Hsp90 clients or regulated by Hsp90 clients, and multiple pre-clinical studies have examined Hsp90’s role in cellular metabolism, primarily as it relates to mitochondrial metabolism^17–20^. To examine the specific effects Hsp90 has on metabolism, one group created an Hsp90-ATPase antagonist specifically directed to mitochondrial Hsp90, and showed that inhibition of mitochondrial Hsp90 depolarized mitochondria and induced cellular apoptosis specifically in tumor cells including those of the breast^21^. Further, inhibition of TRAP1, a member of the Hsp90 chaperone family also caused decreased mitochondrial activity in non-small cell lung cancer^22^. Conversely, another group found that TRAP1 modulates a switch between mitochondrial metabolism and aerobic glycolysis, with over-expression of TRAP1 correlating with a more glycolytic phenotype in murine adult fibroblasts^23^. Though Hsp90 regulates HIF-1 alpha degradation^24,25^, previous studies have not directly examined the relationship between Hsp90 expression, glycolysis, and oxidative phosphorylation in breast cancer.

The central role of Hsp90 in the function of client oncoproteins has fueled investigation of Hsp90 inhibitors (such as luminespib (AUY922), onalespib (AT13887), and ganetespib (STA-9090)) in clinical trials for breast cancer^26,27^ where tumors often acquire resistance to conventional therapies^28–31^. Hsp90 inhibitors are now being actively investigated as potential alternatives or combinational therapies for breast cancers resistant to chemotherapy^32^, trastuzumab^33–35^, radiation therapy^36^, and tamoxifen^37^. In addition to its role in metastatic progression, Hsp90 has become a target to reduce incidence of acquired drug resistance. It has been shown that low dose inhibition of Hsp90 does not induce a heat shock factor response (i.e. co-chaperone protein levels remain unchanged after treatment with the Hsp90 inhibitor) but rather reduces development of tamoxifen resistance in an ER + breast cancer model^37^

Hsp90 has been studied extensively as a therapeutic target in breast cancer, even gaining regulatory approvals for *in vivo* clinical trials for breast cancer. Furthermore, there is a wealth of evidence demonstrating a variety of roles that intracellular and extracellular Hsp90 plays in metastatic progression. The evidence to date suggests a compelling opportunity to leverage the attributes of Hsp90 expression beyond therapeutics with potential applications in breast cancer diagnosis, prognosis, and recurrence risk assessment to name just a few examples. We have previously developed a fluorescently-tethered Hsp90 inhibitor, HS-27, made up of an Hsp90 inhibitor (SNX-5422) tethered via a PEG linker to a clinically ubiquitous tracer, a fluorescein derivative (FITC), that binds to ectopically expressed Hsp90^8^

In this study, we leverage HS-27 to develop a completely non-destructive strategy to assay Hsp90 expression on intact tissue specimens with comparable contrast to *in vivo* drug administration routes, and demonstrate the feasibility of our approach in breast cancer patients. We also demonstrate in *in vitro* and *in vivo* models that expression of ectopic Hsp90 expression increases in breast cancers that are highly glycolytic and that glycolytic cancers are most sensitive to Hsp90 inhibition. This work sets the stage for immediately using Hsp90 to improve outcomes for breast cancer patients without affecting traditional care pathways.

## Results

### Hsp90 is expressed on the cell surface of breast cancer cells

**Figure 1:**
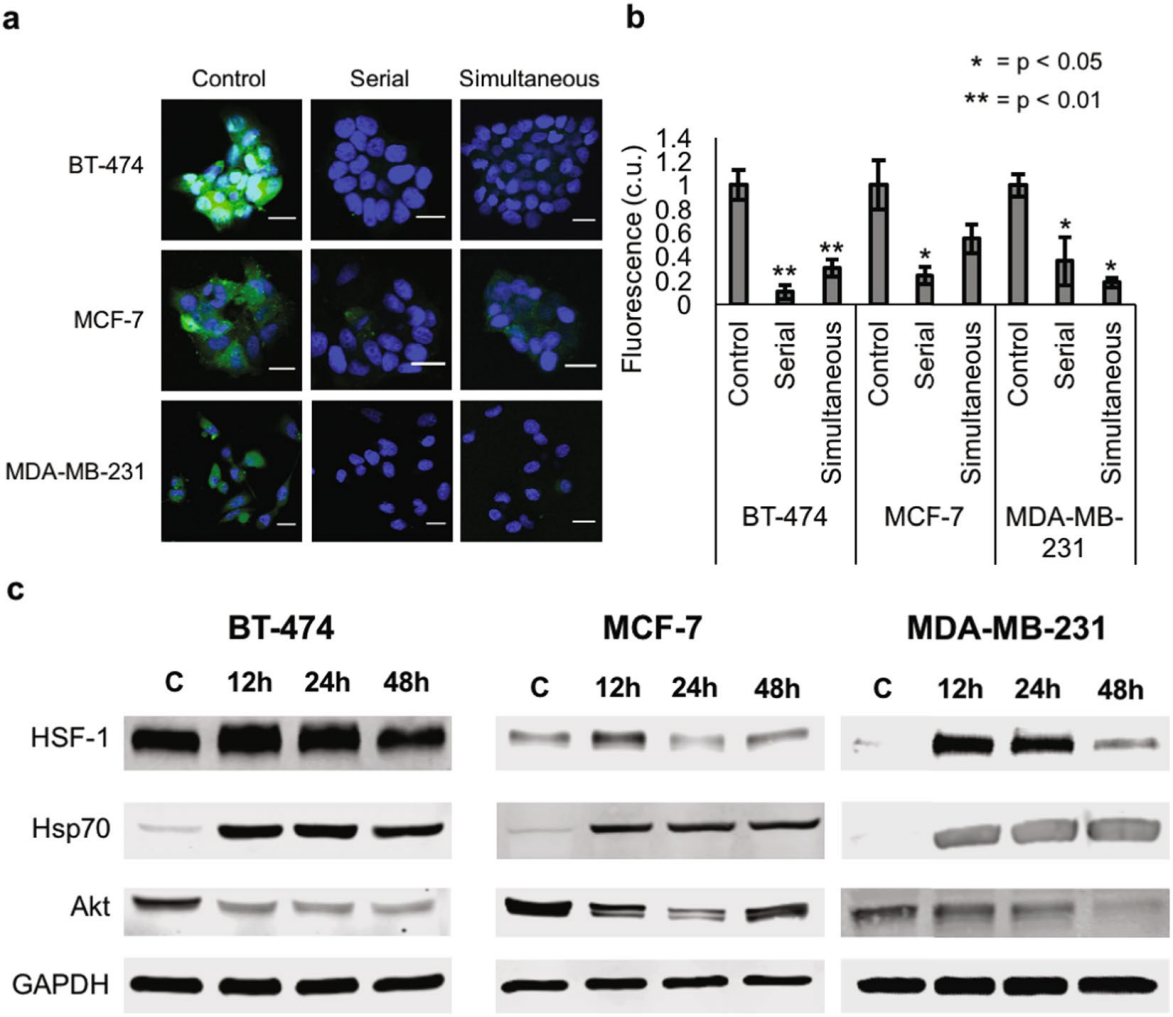
Hsp90 is expressed on the cell surface of breast cancer cells. (**a** and **b**) BT-474 (Her2-overexpressing), MCF-7 (ER + , PR + ), and MDA-MB-231 (triple negative) breast cancer cells were stained with 100 µM HS-27 only for 15 minutes (green, control), serially with 100 µM HS-10 for 14 minutes followed by 100 µM HS-27 for 15 minutes (serial), or simultaneously with 100 µM HS-10 and 100 µM HS-27 (simultaneous) for 15 minutes. DNA is stained in blue with Hoechst 33342. Scale bars are 20 µm. Fluorescence was normalized so that the control images had a value of 1. For all experiments, the sample size was n = 3 per group, and p-values were determined using one-way ANOVA followed by Tukey-Kramer post-hoc testing. (c) Western blotting was performed for HSF-1, Hsp70, and Akt expression in BT-474, MCF-7, and MDA-MB-231 cells after treatment with 100 µM HS-27 for 12, 24, or 48 hours. Cropped western blots are shown; full blots can be found in Supplementary Figure S1. Control cells were incubated with DMSO vehicle. Western blots were performed in triplicate.

To establish that HS-27 can be used as a theranostic agent in breast cancer, we first demonstrated the diagnostic potential of HS-27 in *in vitro* models. Three different receptor subtypes of breast cancer (BT-474 – Her2 + /ER + /PR + , MCF-7 – ER + /PR + , and MDA-MB-231 – TNBC) were incubated with 100 µM HS-27 for 15-minutes, washed thoroughly with PBS to remove unbound HS-27, and imaged with a confocal microscope. Representative images are shown in Fig. 1a. Consistent with previous studies that examined HS-27 uptake in breast cells^8^, 15 minutes of cell incubation resulted in green fluorescence in all three-cell lines, with the lowest fluorescence exhibited by the MCF-7 cells. Next, the non-fluorescent Hsp90 inhibitor HS-10 (SNX-5422) was used to confirm the mechanism of HS-27 uptake by blocking Hsp90 binding sites without contributing to the fluorescence signal. BT-474, MCF-7, and MDA-MB-231 cells were incubated with either HS-27 only for 15-minutes (control), HS-10 for 15 minutes followed by HS-27 for 15 minutes (serial), or with HS-10 and HS-27 together for 15-minutes (simultaneous). Differences in fluorescence quantified from confocal images of HS-27 in the cell lines with different HS-10 perturbations are shown in Fig. 1b. Fluorescence from HS-27 decreased in both serial and simultaneous (p < 0.05) treatment groups compared to control, for BT474 and MDA-MB-231 cell lines confirming that HS-27 uptake decreased when Hsp90 binding sites are blocked with HS-10. The results for MCF-7 were only significant in the case of serial competition (p < 0.05) but not simultaneous competition.

After establishing that HS-27 uptake was ubiquitous across cell lines, the therapeutic potential of HS-27 was investigated by examining protein expression level changes in response to the HS-27 incubation time. BT-474, MCF-7, and MDA-MB-231 cells were treated with 100 µM HS-27 or DMSO control for 12, 24, or 48 hours. Western blots were performed against HSF-1, a transcription factor upregulated during Hsp90 inhibition, Hsp70, a co-chaperone protein upregulated during Hsp90 inhibition, and Akt, an Hsp90 client protein, which degrades during Hsp90 inhibition. Cropped western blots for HSF-1, Hsp70 and Akt expression are shown in Fig. 1c. Full western blots can be found in Supplementary Figure S1. HSF-1 and Hsp70 induction after treatment with HS-27 was seen in all cell lines. In BT-474 and MDA-MB-231 cells, Akt levels were decreased throughout treatment, while in MCF-7 cells, Akt decrease was transient.

### Hsp90 inhibition decreases oxidative and glycolytic metabolism in Her2-overexpressing and triple negative breast cancer

**Figure 2:**
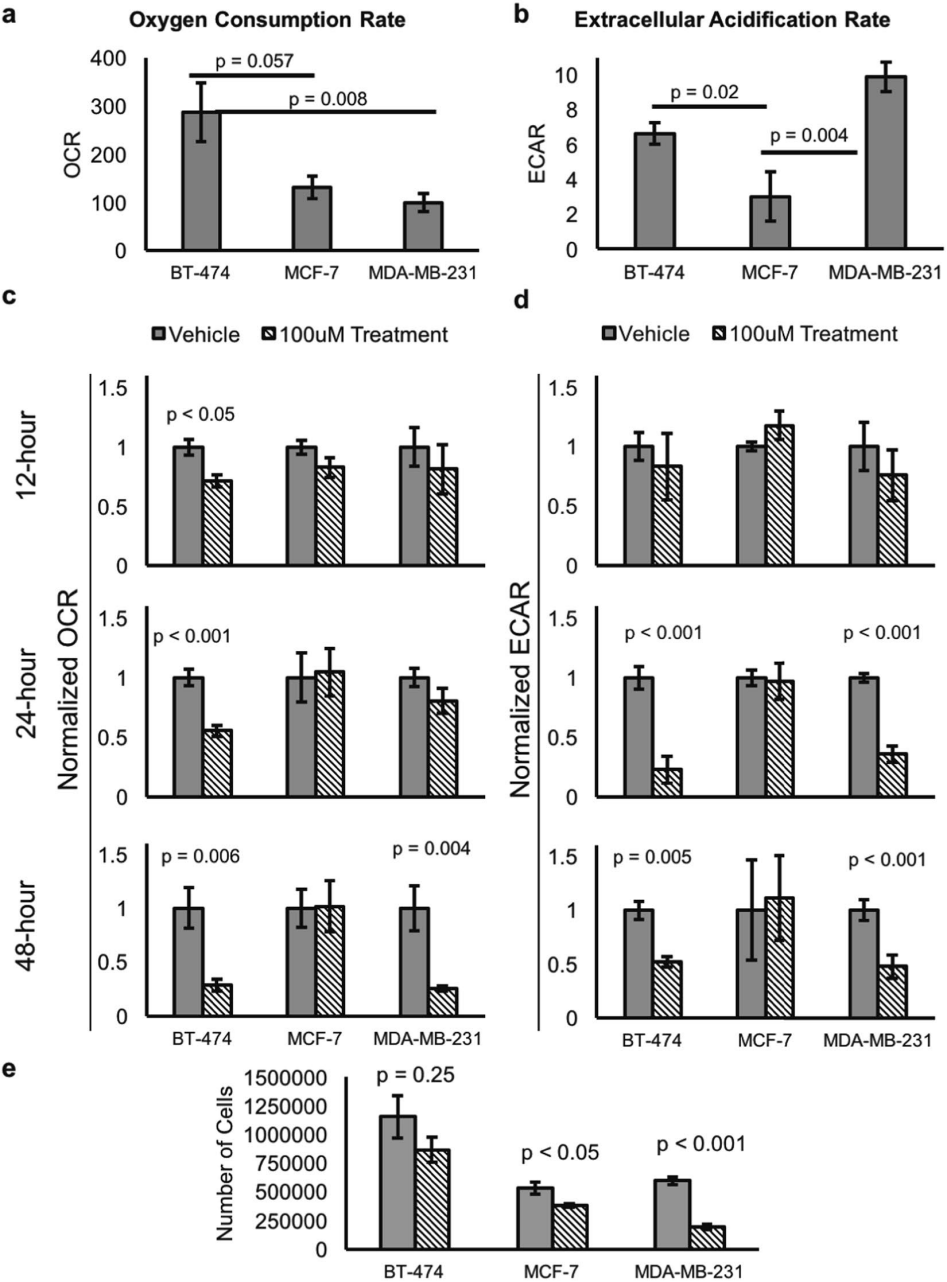
Hsp90 inhibition decreases oxidative and glycolytic metabolism in Her2-overexpressing and triple negative breast cancer. A seahorse extracellular flux analyzer was used to determine the baseline and post-treatment metabolic properties of BT-474, MCF-7, and MDA-MB-231 cells. The endpoints monitored were oxygen consumption rate (OCR, left column) and extracellular acidification rate (ECAR, right column). (**a**) Baseline OCR for each cell line. (**b**) Baseline ECAR for each cell line. (**c**) OCR or (**d**) ECAR for each cell line after 12, 24, or 48-hour treatment with 100 µM HS-27 or DMSO (vehicle). (**e**) MDA-MB-231, MCF-7, and BT-474 cells were plated at uniform density and treated with either 100 µM HS-27 or DMSO control for 48 hours. ANOVA with Tukey-Kramer post hoc testing (n = 6) was used for (**a**) and (**b**), while two-sided t-tests (n = 6) were used for (**c**) and (**d**) (n = 3) for (**e**).

Given that HS-27 inhibition affected Akt expression in breast cancer cells, we investigated both the metabolic profile of breast cancer cells and the metabolic effects of HS-27 treatment using a seahorse extracellular flux analyzer. We compared the oxidative and glycolytic metabolism for each cell line using oxygen consumption rate (OCR) as a surrogate for oxidative metabolism and extracellular acidification rate (ECAR) as a surrogate for glycolytic metabolism. Consistent with previous studies^38^, at baseline BT-474 cells had the greatest oxidative metabolism while MDA-MB-231 had the greatest glycolytic metabolism, as shown in Fig. 2a,b. Next, OCR and ECAR of the breast cancer cells were characterized after treatment with 100 µM HS-27 or DMSO control vehicle for 12, 24, or 48 hours. BT-474 cells showed significant decreases in both OCR and ECAR after 24 and 48 hours of treatment, while MDA-MB-231 cells showed significant decreases in ECAR at both 24 and 48 hours and a significant decrease in OCR only at 48 hours post-treatment. MCF-7 cells did not show significant changes in OCR or ECAR at any of the time points. These results, summarized in Fig. 2c,d, suggest that Hsp90 inhibition at the cell surface affects both oxygen consumption and glycolysis in BT-474 and MDA-MB-231 cells. In addition to reduced basal metabolism, significant decreases (p<0.01) in maximal oxidative and glycolytic metabolism were seen in BT-474 and MDA-MB-231 cells 48 hours post treatment, as shown in Supplementary Figure S2. To demonstrate that the metabolic changes after treatment with HS-27 are functionally relevant, we plated breast cancer cells at uniform density and treated them with either 100 µM HS-27 or DMSO control for 48 hours. We found that treatment with HS-27 resulted in reduced cell growth across all cell lines, though only significant for MDA-MB-231 and MCF-7, as shown in Fig. 2e.

### HS-27 uptake is increased in aggressive breast tumor types *in vivo*

**Figure 3:**
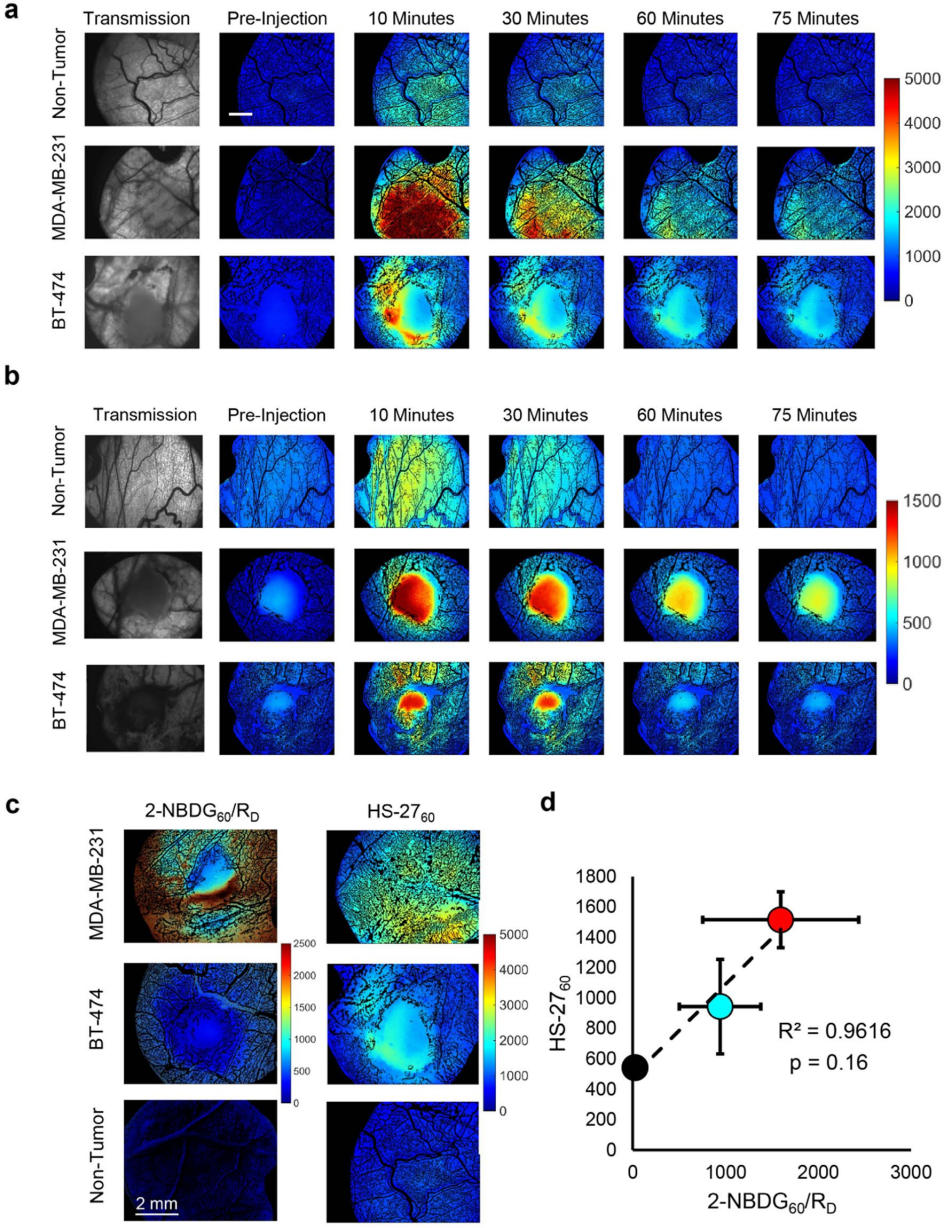
HS-27 uptake is increased in aggressive breast tumor types *in vivo*. (**a** and **b**) Representative transmission and fluorescence images from tumor (MDA-MB-231 and BT-474) and non-tumor bearing window chambers in mice injected with either 20 mg/kg HS-27 (A) or 0.1 mL of 6 mM 2-NBDG (**b**). Transmission images are shown for reference of the tumor location. Scale bar is 2 mm. (**c**) Representative HS-27_60_ or 2-NDBG_60_/R_D_ images for MDA-MB-231 and BT-474 window chambers. (**d**) Average HS-27_60_ and 2-NBDG_60_/R_D_ were plotted against each other, revealing a strong correlation between the two endpoints (R^2^ = 0.96), red = MDA-MB-231, cyan = BT-474, and black = non-tumor window chambers. For HS-27 imaging, n = 3 for all groups. For 2-NBDG imaging, n = 3 for tumor groups, and n = 8 for normal groups.

After establishing that HS-27 treatment was effective on Her2-overexpressing and TNBC cells *in vitro*, we characterized HS-27 uptake and glycolytic metabolism (using a fluorescent glucose analog, 2-NBDG) *in vivo* in a human breast cancer xenograft murine window chamber model. 2-NBDG is a validated reporter of glucose uptake, and we have previously demonstrated that correcting for 2-NBDG delivery, similar to FDG-PET, accurately reports on glucose uptake *in vivo* ^39^. Representative time course fluorescence images for tumor bearing and non-tumor bearing mice following 20 mg/kg HS-27 or 0.1 mL of 6 mM 2-NBDG injection are shown in Fig. 3a,b respectively. To investigate the relationship between surface Hsp90 expression and glucose uptake, we examined HS-27 and delivery corrected 2-NBDG uptake, 60-minutes post injection (HS-27_60_ and 2-NDBG_60_/R_D_). Representative HS-27_60_ and 2-NBDG_60_/R_D_ images of MDA-MB-231 and BT-474 window chambers as well as non-tumor window chambers are shown in Fig. 3c. The averaged HS-27_60_ and delivery-corrected 2-NBDG_60_/R_D_ data points are plotted against each other in Fig. 3d. The images and data reveal a strong association between the HS-27 and 2-NBDG fluorescence intensities (R^2^ = 0.96).

### *Ex vivo* HS-27 application retains contrast between tumor and non-tumor tissue

**Figure 4:**
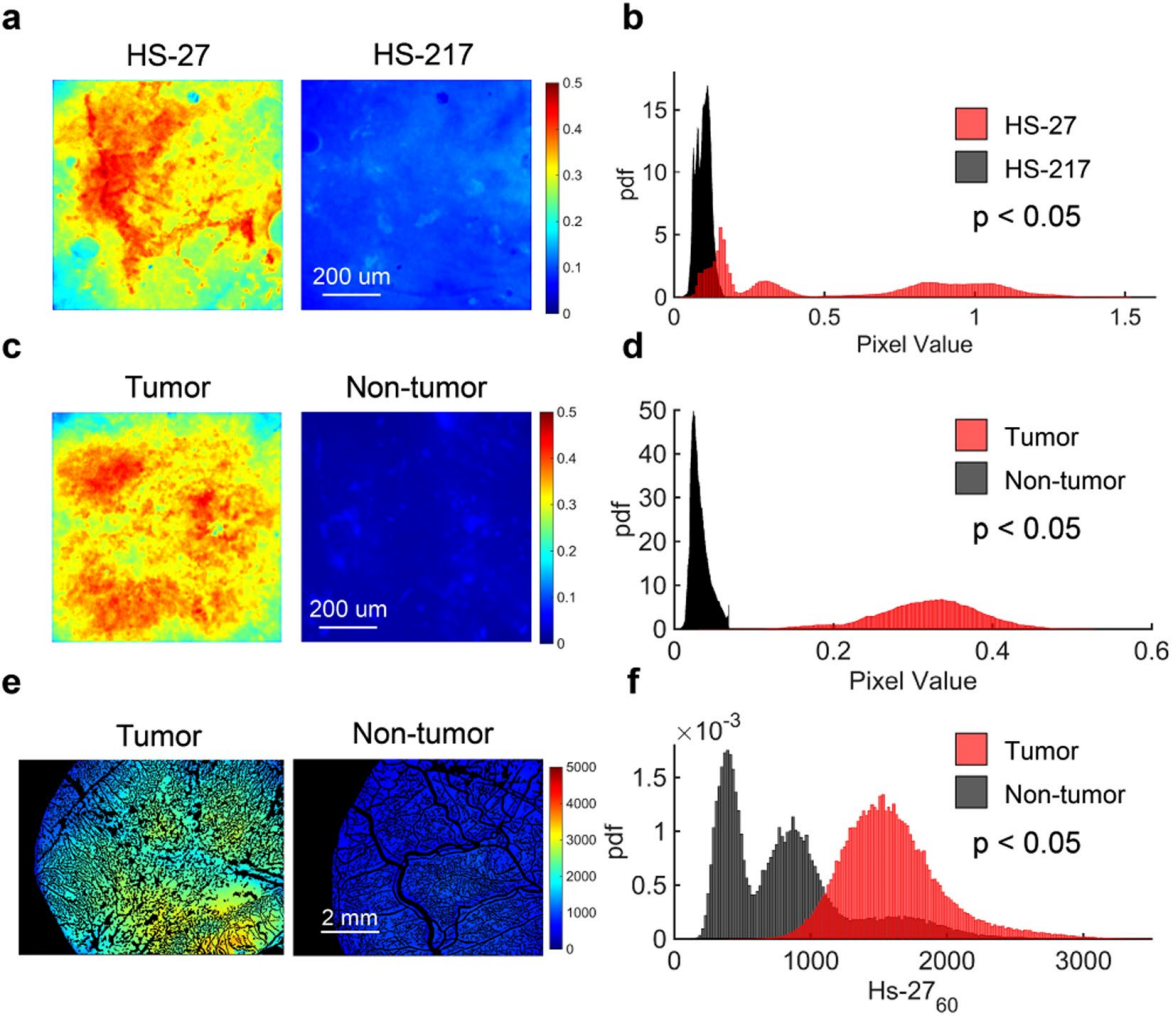
*Ex vivo* HS-27 application retains contrast between tumor and non-tumor tissue. (**a**) Sample images of mouse tumor tissue treated *ex vivo* with 100 µM HS-27 or 100 µM inactivated HS-27 (HS-217) for one minute (**b**) Probability distribution functions (PDFs) of the HS-27 (n = 3) and HS-217 (n = 3) fluorescence values demonstrate significantly greater fluorescence in HS-27 treated samples than HS-217 treated samples. (**c**) Sample images of mouse tumor tissue and mouse skin tissue treated *ex vivo* with 100 µM HS-27 for one minute (**d**) PDFs of the HS-27 fluorescence values for both tumor (n = 3) and non-tumor (n = 3) are shown beneath the images. Fluorescence was significantly greater in tumor than non-tumor samples. (**e**) Sample images of tumor or non-tumor mouse tissue at 60-minutes after i.v. injection of 20 mg/kg HS-27. (**f**) PDFs of HS-27 fluorescence values for both tumor (n = 3) and non-tumor (n = 3) demonstrate significantly greater fluorescence in tumor than non-tumor samples. P-values were calculated using a Kolmogorov Smirnov test.

The quickest path to move a new contrast agent into the clinic is to perform pilot studies on clinical *ex vivo* tissue specimens, eliminating the lengthy IND review process. Before moving to the clinic, we first established that *ex vivo* application of HS-27 to pre-clinical tissue samples retained contrast between tumor and non-tumor tissues. Figure 4 demonstrates it is feasible to detect differences between breast tumors and normal tissues after topical administration of HS-27 to excised specimens. Our first goal was to show HS-27 signal is a result of fluorescence from HS-27 bound to Hsp90 rather than fluorescence from non-specific uptake. We demonstrated this by comparing uptake of active HS-27 to an inactivated version, HS-217. HS-217 is an altered form of HS-27 with additional methyl groups attached to the ligand portion of the molecule, decreasing the ability of HS-217 to bind Hsp90. We have previously shown that the addition of methyl groups in a similar compound decreased affinity for binding to Hsp90^40^. The structure of each HS compound is shown in Supplementary Figure S3. Representative images of an MDA-MB-231 tumor stained *ex vivo* with either 100 µM HS-27 or 100 µM HS-217 for one minute are shown in Fig. 4a. Probability distribution functions (PDFs) of HS-27 and HS-217 pixel intensities were generated for HS-27 and HS-217 treated biopsies obtained from 3 mice as shown in Fig. 4b. HS-27 uptake was significantly greater (p<0.05) than HS-217 uptake seen in the right shift in the HS-27 PDF.

A similar analysis was performed on 3 mice to demonstrate *ex vivo* HS-27 binding is greater in tumor compared to non-tumor adjacent skin. Figure 4c shows representative images of tumor and non-tumor tissue treated with HS-27 for one minute. The soft-tissue on the subcutaneous side of the skin directly over the tumor was chosen as the non-tumor tissue for most direct comparison to *in vivo* treatment in the dorsal skin flap window chamber model. PDFs of HS-27 signal in tumor vs. non-tumor tissue, shown in Fig. 4d, confirm HS-27 uptake is significantly greater in tumor compared to non-tumor tissue. This analysis was also performed on 3 MDA-MB-231 and 3 non-tumor window chambers in mice (shown in Fig. 3 **)** to demonstrate consistency between *in vivo* and *ex vivo* administration routes. Representative HS-27_60_ images are shown for tumor and non-tumor in Fig. 4e,f shows PDFs of tumor and non-tumor HS-27 uptake, demonstrating significantly greater HS-27 uptake in tumor vs. non-tumor tissue. The significant shifts in both the *in vivo* and *ex vivo* PDFs further demonstrate that *ex vivo* HS-27 administration is a viable method for evaluating HS-27 uptake in tumor samples. In fact, contrast (defined as the ratio of tumor fluorescence to non-tumor fluorescence) between tumor and non-tumor tissue was greater than 2 for both *ex vivo* and *in vivo* administration.

### A rapid assay for Hsp90 imaging in clinical breast specimens

**Figure 5:**
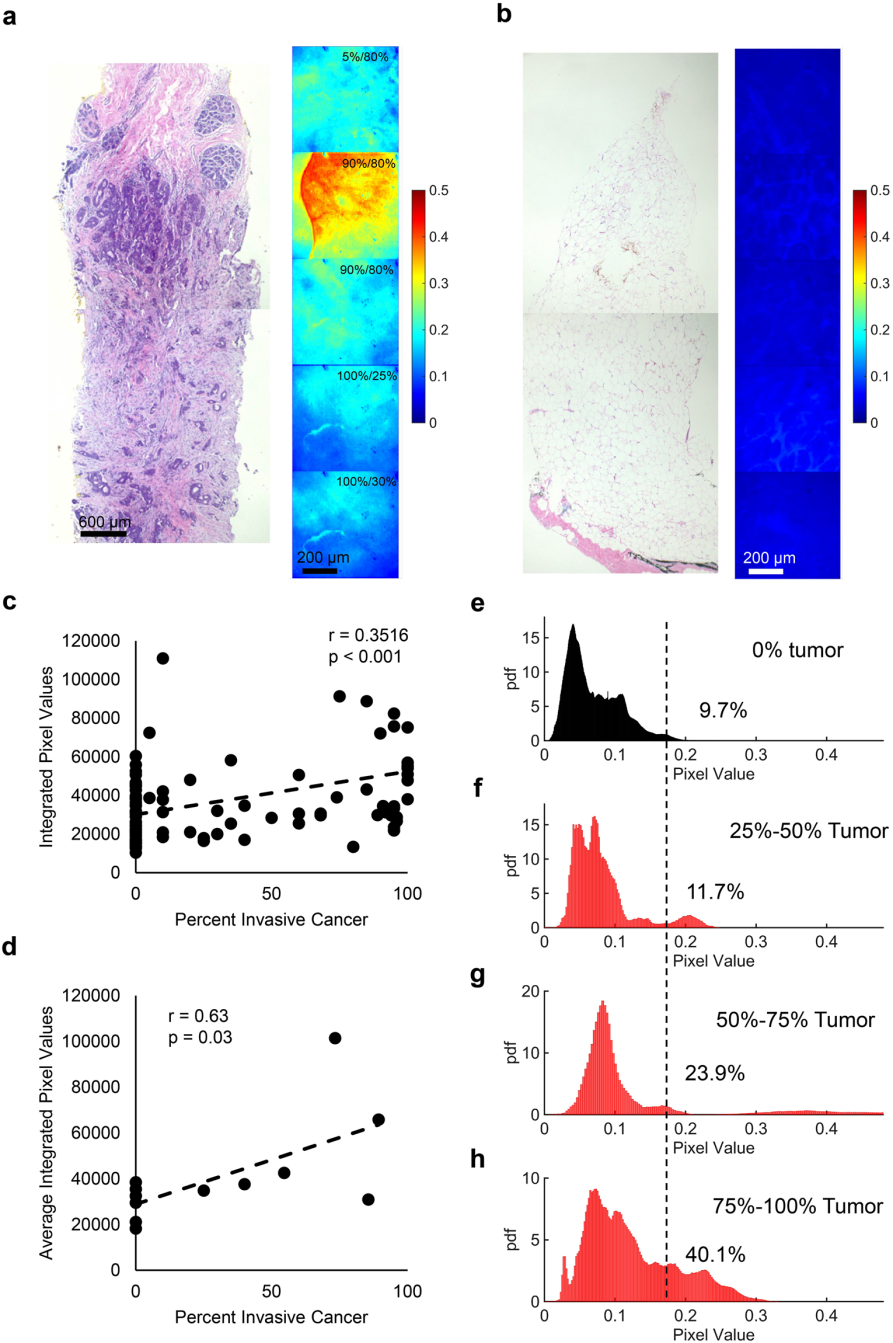
A rapid assay for Hsp90 imaging in clinical breast specimens. (**a** and **b**) Representative fluorescence images of an ultrasound-guided core-needle biopsy treated *ex vivo* with 100 µM HS-27 for one minute for an ER + /PR + tumor (**a**) and non-tumor (**b**) biopsy. Tissue types were determined by a trained pathologist using the histology images shown to the left of the fluorescence images. The top right corner of each tumor image shows the percentage of total tumor involvement in that region along with the percent cellularity. (**c**) Plotting integrated fluorescence as a function of percent cancer at each site (n = 96) demonstrates a positive and significant correlation between the percent invasive cancer and HS-27 uptake at site level. There is one data point outside the graph range that is not shown, but was included in the analysis. (**d**) Plotting the average integrated fluorescence for each biopsy stratified by tumor (n = 6) and non-tumor (n = 6) sites reveals a positive and significant correlation between the percent invasive cancer and HS-27 uptake at biopsy level. (E-H) PDFs created from (**e**) all non-tumor images from all biopsies (n = 45), (**f**) all tumor images from sites with between 25% and 50% tumor involvement (n = 7), (**g**) all tumor images from sites with between 50% and 75% tumor involvement (n = 7), and (**h**) all tumor images from sites with greater than 75% tumor involvement (n = 25). The dashed black line shows the mean tumor fluorescence from all images containing tumor regardless of percent tumor involvement. The number to the right of the dashed black line represents the percentage of pixels falling above the overall tumor mean for each case.

After demonstrating the feasibility of imaging tumor tissue *ex vivo* with HS-27, we performed a pilot clinical study on tissues acquired from 8 ultrasound-guided core needle biopsies. The tumor receptor statuses for patients with tumor containing biopsies were 4 ER + /PR + , 2 ER + /HER2 + , and 1 TNBC. HS-27 fluorescence images were stitched together to form a continuous image along the entire length of the biopsy. A trained pathologist (AH) performed histopathological analysis on each biopsy to identify tissue types along the length of the biopsy. The tissue types were stratified as adipose, fibroglandular, or tumor. For regions of biopsy containing tumor, the percentage of tumor involvement was recorded along with tumor cellularity. A representative continuous biopsy image for a tumor and non-tumor biopsy is shown in Fig. 5a,b respectively. Images of the H&E slide corresponding to this biopsy are shown next to the fluorescence images for comparison. The percentage of tumor involvement and tumor cellularity is shown in the top right corner for only the images containing tumor. Using the site-level pathology data from 96 sites, the integrated fluorescence from each site was plotted against percentage tumor involvement at the site and is shown in Fig. 5c. HS-27 fluorescence weakly but significantly (r = 0.3647, p < 0.001) correlated with percent tumor involvement. The integrated fluorescence for tumor and non-tumor sites within a biopsy were averaged, and plotted against the average tumor percentage, as shown in Fig. 5d, which showed a positive and significant correlation (r = 0.63, p = 0.028).

After demonstrating that HS-27 is taken up specifically by tumor over non-tumor tissue, we wanted to examine the effect of tumor involvement on HS-27 uptake distributions. Images were sorted into four different bins (0% tumor, 25–50% tumor, 50–75% tumor, and 75–100% tumor) based on their tumor involvement as determined by histopathology. PDFs were created for each bin and are shown in Fig. 5e–h. The overall mean from all tumor images (all images containing tumor cells) was calculated and is represented by the black dashed line. We found that only 9.7% of non-tumor pixels have a value greater than the overall tumor mean. A similar calculation at each bin level demonstrated that increasing tumor percentage results in an increase in HS-27 uptake.

## Discussion

Numerous studies have established that Hsp90 is expressed on the surface of tumors cells^8,11,13^. We have previously demonstrated that HS-27 binds exclusively to surface Hsp90^8^ and our current study found that ectopic expression of Hsp90 is ubiquitous across the three main subtypes of breast cancer. We further examined the diagnostic potential of HS-27 and found significantly enhanced contrast in breast tumors compared to normal breast tissue through both *in vivo* and *ex vivo* drug administration routes. Additionally, we developed a method to rapidly assay Hsp90 expression in clinical specimens non-destructively, towards translation to the point of care setting. Finally, we demonstrated in *in vitro* and *in vivo* models that Hsp90 expression increases in glycolytic tumors, and tumors that show increased glycolysis are most sensitive to Hsp90 inhibition. Taken together, our study suggests the potential for HS-27 to be used as a diagnostic and/or prognostic tool in addition to its utility as a therapeutic agent by providing information about the malignant status of tissues as well as biological information indicative of aggressive tumor phenotypes.

Altered cancer metabolism has been extensively studied, and is one of the key hallmarks of cancer^4^, and Hsp90 along with its various isoforms influence metabolism^21–23^. Dennison *et al*. showed that breast tumors span a wide array of metabolic phenotypes, with some tumors exercising a more oxidative phenotype while others favor glycolytic metabolism as their fuel source^38^. Concordant with their findings, our *in vitro* and *in vivo* findings followed the same trend as their *in vitro* findings – the MDA-MB-231 cell line was more glycolytic than oxidative, followed by BT-474 and MCF-7. By examining the relationship between HS-27 and 2-NBDG-uptake we showed that increased ectopic Hsp90 expression is highest in glycolytic tumors. These results demonstrate a direct relationship between increased ectopic Hsp90 expression and aggressive tumor phenotypes, when increased glycolytic metabolism is used as a surrogate for aggressive disease.

We showed that surface Hsp90 inhibition causes decreased metabolism in the more glycolytic MDA-MB-231 and BT-474 breast cancer cells. At baseline these two cell lines were significantly more glycolytic than the MCF-7 cells, though BT-474 cells are known to utilize oxidative metabolism as well. Concordant with previous studies MDA-MB-231 had a significantly lower OCR at baseline than BT-474 cells^38^, suggesting MDA-MB-231 cells are less dependent on oxidative metabolism. Looking at changes in metabolism temporally, BT-474 cells showed significant decreases in both OCR and ECAR on the same timescale, while ECAR decreased in MDA-MB-231 cells prior to decreases in OCR. The early degradation of Akt, which promotes aerobic glycolysis, in MDA-MB-231 cells just 12-hours after HS-27 treatment further explains the early temporal change in ECAR seen in this cell line.

The results from our study suggest that Hsp90 is expressed on the surface of MCF-7 cells, yet inhibition with HS-27 was ineffective at down-regulating glycolysis or oxidative phosphorylation in this cell line. There are numerous barriers between Hsp90 inhibition at the surface of cells and expression of ER within the cell nucleus, which may prevent HS-27 treatment from effectively reducing ER expression in MCF-7 cells. Cell permeant Hsp90 inhibitors, like ganetespib, have therapeutic effect in MCF-7 cells, and one mechanism of action is through the ER/Akt signaling pathway^41^. Ganetespib effectively destabilizes ER, leading to a loss of Akt activity, ultimately resulting in therapeutic potency. In our study, Akt change after HS-27 inhibition was transient and minimal in MCF-7 cells, suggesting that HS-27 does not effectively destabilize the ER, maintaining the integrity of the ER/Akt signaling pathway. ER cancers are typically less aggressive with better clinical outcomes than either TNBC or Her2 + cancers^42^. Given the wealth of studies, mentioned above, implicating Hsp90 in metastatic progression, it is not surprising that MCF-7 cells are less responsive to treatment with HS-27. Despite the lack of change in metabolic activity, HS-27 treatment still significantly reduced MCF-7 cell growth.

Though there was a weakly positive and significant correlation between HS-27 uptake and tumor involvement in our clinical study, we did notice differences in HS-27 signal quantified from sites with the same level of tumor involvement. Several limitations may have caused these differences in signal. Despite attempting to evenly distribute HS-27 across the biopsy sample, inherent human error caused uneven topical HS-27 application and non-uniform PBS rinsing. Additionally, perfect co-registration between fluorescence imaging and histology is not possible as fluorescence images were obtained along the surface of the biopsy whereas histology sections are taken from the center. Because of the difference in depth between imaging and histological sampling, there may be slight differences in tissue composition between the imaging and histology sites. Another source of error results from contact between the HRME probe and the sample in order to take an image. Pressure from tissue contact may deform the tissue making the imaging depth different between adjacent images, further limiting exact co-registration with histology. Finally, as with any optical technology, contrast is limited by presence of any background signal. One biological source of background could be auto fluorescence from redox imaging targets naturally occurring in tissue like NADH and FAD^43^, which could add a second source of fluorescence signal not emanating from HS-27.

To address these shortcomings in future studies, we will adapt our system for non-contact imaging to eliminate pressure application on the tissue. We also will implement an automated strategy to stain and rinse HS-27 to ensure even HS-27 application and washing across the entire sample. Additionally, we will devise a method to cut the biopsy in half to examine cross-sectional areas for the presence of tumor cells, as well as incorporate a near-infrared version of HS-27 to reduce background signal from auto fluorescence, while simultaneously reducing loss of signal caused by scattering. The combination of our new imaging system and fluorescent agent will facilitate translation of this technology into the clinical setting.

Our next steps are to extend our studies to image samples from different breast cancer subtypes and demonstrate the utility of our technology in both the diagnostic and surgical settings. In the diagnostic biopsy setting, patients require sampling of multiple cores to ensure that the suspicious lesion was captured by a biopsy, increasing patient discomfort and care time. The highly specific nature of HS-27 binding to tumor tissue would allow a radiologist to instantly test each core as it is removed to check for disease in the biopsy. By assessing biopsy samples during the procedure, the HS-27 results could improve the radiologists’ confidence that lesions have been adequately sampled, particularly for highly suspicious lesions that stain positive for HS-27.

In the margin assessment setting, HS-27 could be topically applied to the tumor margin after excision to provide intra-operative feedback to the surgeon regarding the malignant status of the margin. Given that between 20% and 40% of patients undergoing breast conserving surgery have a positive tumor margin requiring re-excision^44^, a method to reduce re-excision rates would increase the number of primary surgeries surgeons can perform, and would reduce the burgeoning costs of repeat visits and interventions on an already depleted health care system.

## Materials and Methods

### Cell Culture

BT-474, MCF-7, and MDA-MB-231 breast cancer lines were used in the cell study. All cells were acquired from the American Type Culture Collection and cultured under standard conditions free of contamination at 37 °C and 5% CO_2_. BT-474 cells were cultured in RPMI-1640 (L-glutamine) medium while MCF-7 and MDA-MB-231 cells were cultured in MEME medium. All medium was supplemented with 10% FBS, 2.8 mL 45% glucose solution per 500 mL media (BT-474), 1% sodium pyruvate, 1% non-essential amino acids (MCF-7 and MDA-MB-231), 0.01 mg/ml insulin (BT-474 and MCF-7) and 1% penicillin-streptomycin. All cells were used for experiments within two months of first passage.

### Live Cell Imaging

To monitor differences in HS-27 uptake between cell lines, BT-474, MCF-7, and MDA-MB-231 cells were incubated with 100 uM HS-27 (chosen based on previous studies with HS-27^8^) for fifteen minutes at 37 °C by adding HS-27 dissolved in DMSO directly to the cellular media. To confirm the mechanism of HS-27 uptake by serially or simultaneously blocking Hsp90 binding sites, BT-474, MCF-7, and MDA-MB-231 cells were incubated with either 100 µM HS-27 only (control), 100 uM HS-10 followed by 100 µM HS-27 (serial), or simultaneously with 100 µM HS-10 and HS-27. The final concentration of DMSO in the media was 1% or less to not affect cell permeability. Cells were rinsed with PBS to remove any unbound HS-27 and fresh media with Hoescht 33342 nuclear stain was added to the plate.

Live cell imaging was performed using a Zeiss 780 upright confocal microscope with sub cellular level resolution at the Duke University Light Microscopy Core Facility. Hoescht 33342 was excited with a 405 nm laser diode, and emission was collected from 410–420 nm. HS-27 was excited using a 488 nm argon laser source, and emission was collected from 500–550 nm. Collected raw images were processed using CellProfiler^45^. All results are shown as the average of the mean fluorescence intensity from at least three different fields of view across two independent imaging sessions ± standard error (SE).

### Western Blotting

BT-474, MCF-7, and MDA-MB-231 cells were treated with either 100 µM HS-27 or DMSO control (vehicle) for 12, 24, or 48 hours, after which they were harvested, lysed, and analyzed with western blotting. Western blot membranes were incubated with primary antibodies HSF-1, Hsp70, Akt, or GAPDH, all acquired from CellSignaling. Fluorescent secondary antibodies (Li-Cor) were used for signal detection.

### Seahorse Assay

A mitochondrial stress test on a Seahorse XF24 Extracellular Flux Analyzer (Seahorse Biosciences, Massachusetts USA) was used to investigate the metabolic parameters of BT-474, MCF-7, and MDA-MB-231 cells treated with either 100 µM HS-27 or DMSO (control) for 12, 24, or 48 hours. Oxygen consumption rate (OCR) was measured as the change in oxygen dissolved in the cellular media over time and serves as a surrogate measurement for oxidative metabolism. Extracellular acidification rate (ECAR) was measured as the change in free proton concentration in the cellular media over time and serves as a surrogate measurement for glycolytic metabolism. For studies looking at metabolic response to HS-27 treatment, data was normalized to DMSO-treated cells. Results are shown as the mean ± SE of 6 wells across two independent experiments.

### Cell proliferation assay

To determine whether the metabolic changes in breast cancer cells were functionally relevant, BT-474, MCF-7, and MDA-MB-231 cells were plated at uniform density and incubated with either 100 µM HS-27 or DMSO control for 48-hours. After treatment, cells were harvested and counted to identify differences in proliferation rates. Results are shown as the mean ± SE number of live cells in culture after 48-hours from three independent experiments (n = 3).

### Animal studies

All animal experiments were performed in accordance with protocols approved by the Duke University Institution for Animal Care and Use Committee. Animals were housed on-site with continual access to food and water under normal 12-hour light/dark cycles.

### Dorsal skin flap window chamber model

In order to investigate differences in HS-27 uptake between non-tumor and tumor-bearing mice, nine female athymic nude mice had titanium window chambers implanted on their dorsal skin flap while under anesthesia (i.p. injection of 100 mg/kg ketamine and 10 mg/kg xylazine)^46^. Three mice had 50 µl of serum-free medium containing 5 × 10^5^ MDA-MB-231 cells injected under fascia tissue for tumor development. For three mice, a 3 mm diameter core biopsy from a BT-474 flank tumor grown in a donor mouse was cut into 1 mm thick pieces and implanted under the fascia tissue. For all tumor windows, the exposed tissue was covered with cover glass, and tumors were allowed to grow for 6–10 days prior to hyperspectral and fluorescence imaging. Non-tumor mice (n = 3) received no injection, and were imaged on the same timeline as the tumor groups.

To investigate the relationship between tumor metabolism and HS-27 uptake, fourteen additional female athymic nude mice had titanium window chambers implanted on their dorsal skin flap using the same procedure as above. MDA-MB-231 tumor-bearing windows (n = 3) had a 3 mm diameter core biopsy from a MDA-MB-231 flank tumor grown in a donor mouse cut into 1 mm thick pieces implanted under the fascia tissue before covering exposed tissue with cover glass. The same process was followed for BT-474 tumor bearing windows (n = 3). Non-tumor windows (n = 8) received only saline. Tumors were allowed to incorporate into the surrounding tissue for 6–10 days prior to hyperspectral and fluorescence imaging.

### Hyperspectral and fluorescence imaging of *in vivo* HS-27 and 2-NBDG uptake

All *in vivo* imaging was performed on a Zeiss Axioskop 2 microscope provided by the Optical Imaging Facility at the Duke Cancer Institute. The imaging system has been previously described in detail^39^. Briefly, a 2.5x objective (NA = 0.075) was used for all vascular (trans-illumination) and fluorescence imaging. All imaging was performed using a Zeiss FluorArc mercury lamp as the source. For hyperspectral imaging, trans-illumination images were captured from 520–620 nm in 10 nm increments and corrected using calibration images of a neutral density filter (ND = 2.5) taken across the same wavelength range. Fluorescence imaging of HS-27 and 2-(N-(7-Nitrobenz-2-oxa-1,3-diazol-4-yl)Amino)-2-Deoxyglucose (2-NBDG) was performed using a 470 ± 20 nm excitation and 525 ± 5 nm collection with an integration time of 200 ms for HS-27 and 800 ms for 2-NBDG. A fluorescence image of a 90.8 nM rhodamine solution was used to correct for variations in lamp intensity between days.

Mice were under anesthesia using vaporized isoflurane (1–1.5% v/v) in room air throughout each imaging session. First, vascular images were captured using the hyperspectral imaging technique described above. Next, a background fluorescence image was captured prior to tail vein injection of either 20 mg/kg HS-27 dissolved in DMSO or 100 µl of 6 mM 2-NBDG dissolved in PBS. Fluorescence images were captured for 75-minutes post injection.

MATLAB (MathWorks) was used for all *in vivo* image processing. HS-27_60_ is HS-27 uptake 60-minutes post injection, and is defined as the intensity at each non-vascular pixel within the fluorescence image taken 60-minutes post injection. Image processing for 2-NBDG imaging is similar to HS-27 and has been previously described^39^.

### *Ex vivo* imaging and analysis of HS-27 uptake in pre-clinical biopsy samples

MDA-MB-231 tumors were established in six to eight week old athymic nude mice by subcutaneous injection of 4 × 10^6^ MDA-MB-231 cells in 100 µL serum-free media. Tumors were allowed to grow to a volume of at least 200 mm^3^ prior to tumor biopsy.

Mice were under anesthesia using vaporized isoflurane (1–1.5% v/v) in room air during biopsy, and euthanized immediately after biopsy was complete. A 14-gauge Achieve programmable automatic biopsy system was used to extract biopsies from each tumor. To establish that HS-27 uptake is a result of binding to Hsp90 on the cell surface, uptake of HS-27 in tumor biopsy was compared with HS-217 (an inactivated version of HS-27 that does not bind Hsp90) uptake in three mice. Two biopsies were taken from each tumor. One biopsy was stained topically with 100 µM HS-27 while the other was stained topically with 100 µM HS-217. After one minute of agent exposure, unbound HS-27 or HS-217 was washed off using PBS. Images were acquired of each biopsy using a high-resolution microendoscope (HRME).

To establish tumor specific uptake of HS-27, the same procedure described above was performed on n = 3 mice. For these mice, one biopsy was taken per mouse along with the skin directly adjacent to the tumor. The soft tissue on the subcutaneous side of the skin was used as a control to compare contrast between tumor and non-tumor tissue with *in vivo* imaging. The tumor biopsies and skin samples underwent the same HS-27 incubation and HRME imaging described above.

All HRME images were processed using MATLAB (MathWorks) to create PDFs for statistical analysis.

### *Ex vivo* imaging and analysis of HS-27 uptake in clinical biopsy samples

All clinical imaging was performed in accordance with Duke IRB approved protocol number Pro00008003. After giving informed consent, twelve adult patients undergoing standard of care ultrasound guided needle core biopsy have been enrolled in our study. Our imaging platform failed for one patient leaving eleven for analysis. Of these eleven patients, three have been excluded from analysis as they were used for protocol optimization.

Our biopsy imaging procedure and high resolution microscope (HRME) have been previously described in detail^47,48^. Briefly, immediately after tissue retrieval using USGCNB, 100 µM HS-27 was applied topically and allowed to stain the tissue for one minute. After staining, the biopsy was thoroughly rinsed with PBS to remove any unbound HS-27, and the HRME was used to collect images along the length of the biopsy in 1 mm increments using a translation stage. After completion of imaging, each half of the biopsy was inked in either orange or green ink to orient the tissue for histopathological analysis, fixed in formalin, sectioned, and stained with hematoxylin and eosin (H&E). Beginning at the center of the biopsy where the green and orange ink meet, the pathologist examined the biopsy in 1 mm increments to determine what percentage of each segment was composed of tumor, adipose, and/or fibroglandular tissue. For segments containing tumor, the pathologist provided the tumor cellularity, or the percentage of the area occupied by tumor that was composed of tumor cells versus tumor associated stroma.

Images were processed using MATLAB (MathWorks). If the image contained invasive ductal carcinoma (IDC), it was classified as tumor. Images that did not contain tumor were classified as non-tumor. Images were binned according to the amount of tumor present based on histology, either as 0% tumor, 25–50% tumor, 50–75% tumor, or 75–100% tumor.

### Statistical analysis

A two-sided student’s t-test was used for experiments comparing only two groups. Experiments with three or more groups were compared using an analysis of variance (ANOVA) with Tukey post hoc testing to calculate p-values. PDFs were compared using a Kolmogorov Smirnov test. Pearson’s linear correlations were used to calculate correlation coefficients. Comparisons were considered significant on a 95% confidence interval with a p-value of 0.05 or less. All statistical testing was performed using the Statistics Toolbox in MATLAB (MathWorks).

### Data availability statement

The datasets generated during and/or analyzed during the current study are available from the corresponding author on reasonable request.

## Electronic supplementary material

Supplementary Figures
