# Supplementary material for: Leveraging ectopic Hsp90 expression to assay the presence of tumor cells and aggressive tumor phenotypes in breast specimens
Source: Sci Rep. 2017 Dec 13;7:17487. doi: 10.1038/s41598-017-17832-x (PMC5727497; doi:10.1038/s41598-017-17832-x)
Supplement: Supplementary file 1 — Supplementary Figures [file 41598_2017_17832_MOESM1_ESM.pdf]

Supplementary Materials

Leveraging ectopic Hsp90 expression to assay the presence of tumor cells and aggressive tumor phenotypes in breast specimens

Authors: Brian Crouch[1*], Helen Murphy[1], Stella Belonwu[2], Amy Martinez[1], Jennifer Gallagher[3], Allison Hall[4], Mary Scott Soo[5], Marianne Lee[1], Philip Hughes[6], Timothy Haystead[6], Nirmala Ramanujam[1,6]

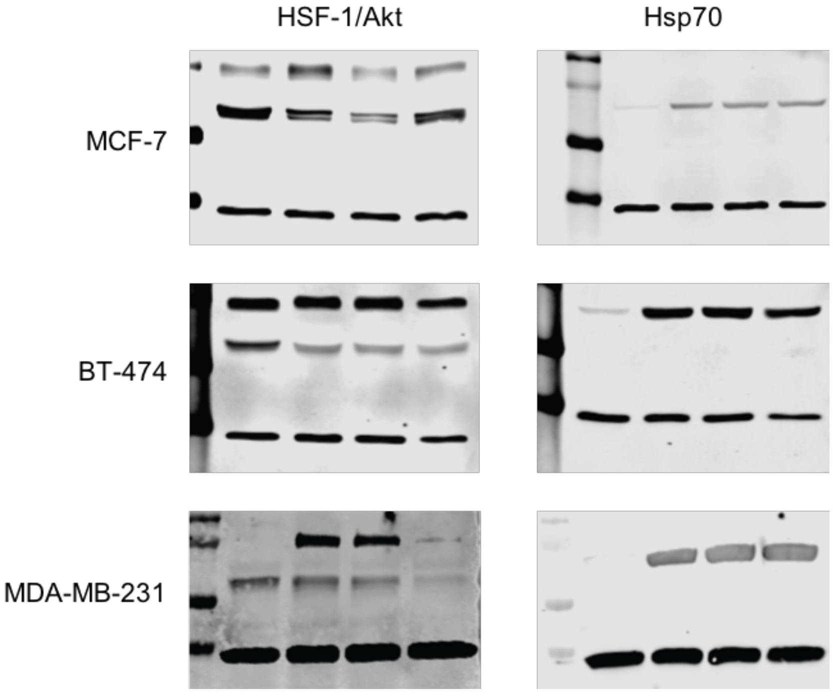

Supplementary Figure S1: Full western blots from HS-27 treatment. Full western blots from HS-27 treatment are shown for MCF-7, BT-474, and MDA-MB-231. Blots on the left are for HSF-1 and Akt with GAPDH loading control. Blots on the right are for Hsp70 with GAPDH loading control.

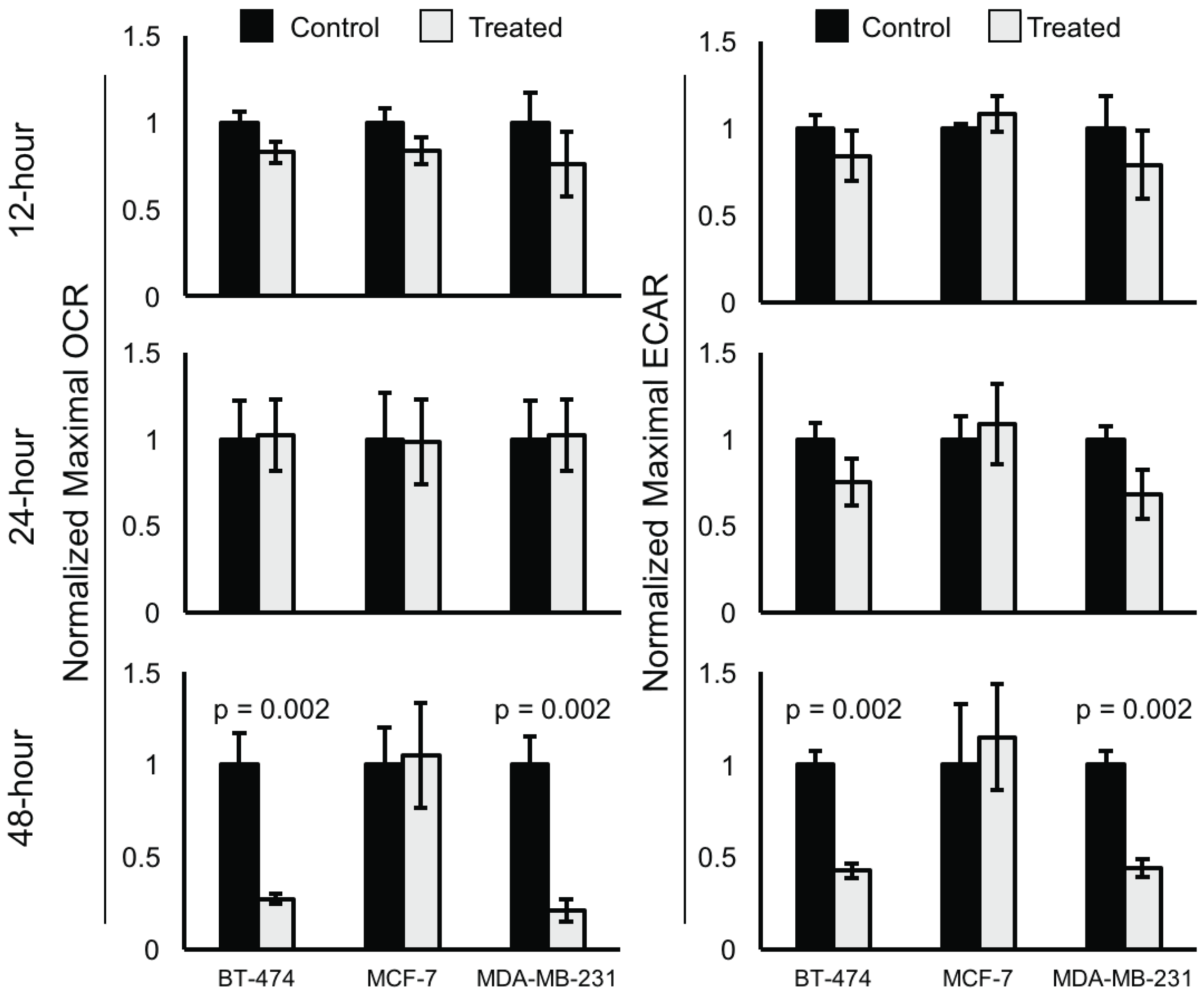

Supplementary Figure S2: Hsp90 inhibition affects maximal metabolism in Her2-overexpressing and triple negative breast cancer 48-hours post treatment. A seahorse extracellular flux analyzer was used to determine the post-treatment metabolic properties of BT-474, MCF-7, and MDA-MB-231 cells. Maximal OCR and maximal ECAR for each cell line after 12, 24, or 48-hour treatment with 100 μM HS-27 or DMSO (vehicle) are shown in the left and right columns respectively. Two-sided t-tests (n=6) were used to determine significance and are shown where applicable.

a

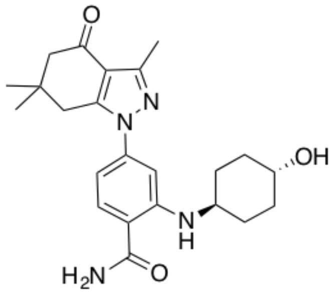

HS-10

b

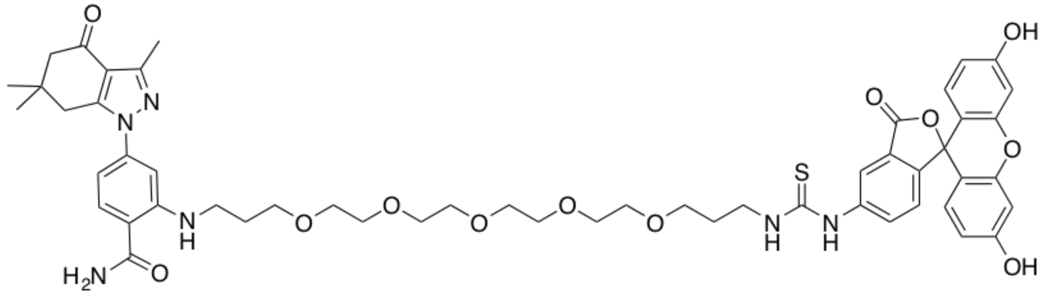

HS-27

c

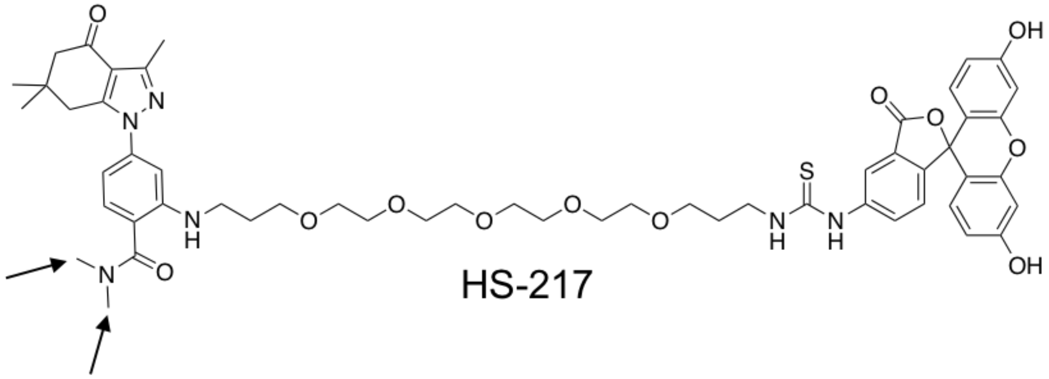

HS-217

Supplementary Figure S3: Chemical structures of Hsp90 inhibitors. Chemical structures are shown for the parent Hsp90 inhibitor HS-10 (A), FITC tethered Hsp90 inhibitor HS-27 (B), and de-activated form of HS-27, HS-217, (C). HS-27 and HS-217 differ only by replacing the two hydrogen atoms of the amino group in HS-27 with two methyl groups, indicated by the black arrows.
